# Neutrophilic Bronchial Inflammation Correlates with Clinical and Functional Findings in Patients with Noncystic Fibrosis Bronchiectasis

**DOI:** 10.1155/2015/642503

**Published:** 2015-12-27

**Authors:** Federico L. Dente, Marta Bilotta, Maria Laura Bartoli, Elena Bacci, Silvana Cianchetti, Manuela Latorre, Laura Malagrinò, Dario Nieri, Maria Adelaide Roggi, Barbara Vagaggini, Pierluigi Paggiaro

**Affiliations:** Cardiothoracic and Vascular Department, University of Pisa, Via Paradisa 2, 56124 Pisa, Italy

## Abstract

*Background*. Neutrophilic bronchial inflammation is a main feature of bronchiectasis, but not much is known about its relationship with other disease features.* Aim*. To compare airway inflammatory markers with clinical and functional findings in subjects with stable noncystic fibrosis bronchiectasis (NCFB).* Methods*. 152 NFCB patients (62.6 years; females: 57.2%) underwent clinical and functional cross-sectional evaluation, including microbiologic and inflammatory cell profile in sputum, and exhaled breath condensate malondialdehyde (EBC-MDA). NFCB severity was assessed using BSI and FACED criteria.* Results*. Sputum neutrophil percentages inversely correlated with FEV1 (*P* < 0.0001; rho = −0.428), weakly with Leicester Cough Questionnaire score (*P* = 0.068; rho = −0.58), and directly with duration of the disease (*P* = 0.004; rho = 0.3) and BSI severity score (*P* = 0.005; rho = 0.37), but not with FACED. Sputum neutrophilia was higher in colonized subjects,* P. aeruginosa* colonized subjects showing greater sputum neutrophilia and lower FEV1. Patients with ≥3 exacerbations in the last year showed a significantly greater EBC-MDA than the remaining patients.* Conclusions*. Sputum neutrophilic inflammation and biomarkers of oxidative stress in EBC can be considered good biomarkers of disease severity in NCFB patients, as confirmed by pulmonary function, disease duration, bacterial colonization, BSI score, and exacerbation rate.

## 1. Introduction

Noncystic fibrosis bronchiectasis (NCFB) is a disease characterized by permanently dilated airways due to bronchial wall structural components destruction, as a result of bronchial inflammation caused by recurrent or chronic infections [1]. Clinical and functional features of NCFB are chronic cough, at times with haemoptysis, increased sputum production due to impaired mucus clearance, dyspnoea, and frequent bacterial colonization.

Neutrophils play a key role in the development and progression of bronchiectasis. Bronchial biopsies in patients with bronchiectasis have demonstrated tissue neutrophilia, a mononuclear cell infiltrate composed mainly of CD4+ T cells and CD68+ macrophages, and increased expression of IL-8 and other chemokines [2]. Neutrophil massive recruitment into the airways in response to infective or inflammatory triggers results in proteolytic enzymes such as neutrophil elastase (NE) and matrix metalloproteinases (MMP) release, leading to airway matrix destruction [3]. The damage of the epithelial layer is responsible for reduced mucociliary clearance efficiency, leading to bacterial colonization and perpetuating the vicious circle “bacterial load-inflammation-airway damage.”

Systemic inflammation as demonstrated by increased blood neutrophils and plasma cytokines (like TNF-alfa) has been measured in patients with NCFB, with some relationship with disease severity and bacterial colonization [4]. Despite the relevant role the neutrophilic inflammation is believed to have in the progression of the disease, few studies have evaluated the relationship between sputum inflammatory biomarkers and clinical or functional findings in patients with bronchiectasis [5–7]. In addition, very few data are reported on the measurement of oxidative stress biomarkers in exhaled air in this kind of patients [8].

The aim of this study was to evaluate the level of bronchial inflammation in subjects with stable NCFB and to compare it with clinical and functional findings in the attempt to understand its role in NCFB severity and progression. We used sputum inflammatory cell counts for confirming and extending previous data from the literature and EBC-MDA as a new tool in the correlation between biomarkers and clinical and functional findings.

## 2. Patients and Methods 

We performed a cross-sectional study on 152 patients with noncystic fibrosis bronchiectasis (NCFB) presenting to our pulmonary unit. Criteria for entering in the study were the following: (a) NFCB diagnosis according to high resolution computed tomography (HRCT) [9], (b) absence of clinical, radiological, and laboratory tests suggestive of cystic fibrosis, and (c) stable phase of the disease (no acute exacerbations or antibiotic use in the previous 4 weeks). Diagnosis and assessment of NCFB have been done according to BTS Guidelines [10]. Patients with long-lasting clinical history of COPD (confirmed by spirometry) associated with smoking habit and bronchiectasis associated with interstitial lung diseases were not included.

In the same day, patients underwent complete spirometric evaluations (including measurement of diffusing capacity and bronchodilator reversibility), arterial blood gas analysis, Leicester Cough Questionnaire (LCQ, in 62 out of 152 patients) [11], assessment of the number and severity of comorbidities, assessment of the number of acute exacerbations in the last year (defined as increase in cough and/or sputum requiring a short course of antibiotics, in few cases also as fever and/or chest pain), sputum collection for bacterial colonization and inflammatory cell analysis, exhaled breath condensate (EBC) collection for malondialdehyde (MDA) assay, and exhaled Nitric Oxide (NO) measurement. Information about the number and the severity of exacerbations in the last year was collected. The aetiology of bronchiectasis was derived from the clinical history, CT features and from a set of blood analyses (including serum immunoglobulin, standard rheumatologic blood tests, and blood cell counts), while more detailed blood tests (like extensive rheumatologic evaluation, serum IgE or IgG for* Aspergillus*, and nasal biopsy) were limited to patients with some clinical suspect of specific diseases (like mucociliary dyskinesia, bronchopulmonary aspergillosis, and rheumatologic disorders). AAT1 was not systematically measured. Localization, extent, and type of bronchiectasis and presence of any inflammatory impairment/alteration due to other/different diseases were evaluated by HRCT performed in the last year before the study assessment [1, 12]. NCFB severity was assessed by both FACED score and Bronchiectasis Severity Index (BSI) [13, 14].

Pulmonary function testswere performed using Medical Graphics equipment (Elite Series Plethysmography, Medical Graphics, Saint Paul, USA), according to the ATS recommendations [15]. Arterial blood sample for the measurement of PaCO2 and PaO2 was taken in sitting position. Pharmacologic treatment with bronchodilators and/or inhaled corticosteroids (ICS) was stopped 2 days before spirometry, while long-term azithromycin treatment was withdrawn 4 weeks before the study day.

Informed signed consent was obtained from each patient. As an observational study, the study protocol had been notified to the local Ethic Committee of our University Hospital.

### 2.1. Sputum Induction and Analysis

Sputum collection and analysis were carried out according to a standardised protocol. In patients with spontaneous sputum production (*N* = 35), samples were collected for bacteriology and inflammatory cell count, after mouth rinsing with normal saline. In patients who did not expectorate spontaneously (*N* = 74), sputum production was induced with hypertonic saline solution (HS: NaCl 4.0% w/v) (2.8 mL/min output; Sirius, Technomed, Firenze, Italy), after inhaled salbutamol pretreatment. Nebulization was stopped after 15 min or earlier if FEV1 fell by 20% or more from baseline values [16].

Bacterial assessment of the spontaneous or induced sputum was performed according to the current standard laboratory methods. Gram-positive and Gram-negative bacteria, TB and non-TB mycobacteria, and fungi were searched.

For inflammatory cell counts, sputum samples were processed within two hours from collection; more dense portions were selected and processed as previously described [17]. At least 350 inflammatory cells were counted and macrophage, lymphocyte, neutrophil, and eosinophil values were expressed as percent of total inflammatory cells. Slides with cell viability <50% or an amount of squamous cells such that 350 inflammatory cells could not be counted were considered inadequate and discarded. Our reproducibility for sputum inflammatory cell count was previously assessed: excluding lymphocytes (RI: 0.15), it was considered as satisfactory: RI was 0.80 for macrophages, 0.85 for neutrophils, and 0.82 for eosinophils [18].

### 2.2. Exhaled Breath Condensate (EBC) Collection and MDA Analysis

EBC was collected by cooling exhaled air with a specially designed condenser (ECoScreen, Jaeger, Wurzburg, Germany). Subjects breathed tidally for 15 min through a two-way nonrebreathing valve by which inspiratory air and expiratory air are separated, and saliva is trapped [19]. The condensate obtained was immediately stored at −30°C until analysis. MDA in EBC was quantified, according to the method described by Lärstad et al. [20], using High-Performance Liquid Chromatography (HPLC) (Waters 1525) with fluorescence detector (Multi √ Fluorescence Detector, Waters 2475), after derivatization with thiobarbituric acid (TBA). The MDA-TBA adduct was detected using excitation and emission wavelength of 532 nm 553 nm, respectively. Our limit of detection was 6 nm/L, the intra- and interassay reproducibility were 0.9 and 10.4%, respectively, and the recovery was 96% [21].

### 2.3. Exhaled Nitric Oxide (NO) Measurement

NO was measured in exhaled air, using a Nitric Oxide Analyzer (NOA 280, Sievers Instruments, Inc., USA). Patients performed a single slow exhalation (30–45 sec) through a mild resistance, while maintaining expiratory flow of about 50 L/min, and NO concentration at mouth level was registered all along the expiration phase. At least three acceptable maneuvers with NO variability lower than 10% were obtained, and the highest value was considered [22].

### 2.4. Statistical Analysis

Pulmonary function parameters are expressed as mean ± SD. Biological data are expressed as median and range. Differences between two groups were tested by means of *t*-test for normally and Mann-Whitney test for nonnormally distributed variables. For analysis of larger groups, ANOVA test, for normally distributed variables, and Kruskal-Wallis test, for nonnormally distributed variables, were used.

Correlations were evaluated by means of Spearman's rank correlation test. A *P* value lower than 0.05 was considered as significant.

## 3. Results 

### 3.1. Characteristics of the Examined Patients

We studied 65 males and 87 females with NCFB, with a mean age of 62.6 years. Main demographic and clinical characteristics are reported in Table 1. Bronchiectasis was diffuse in 84% and localized in 10% of patients; 77% showed cylindric, 12% cystic, and 5.3% varicose bronchiectasis (in the remaining patients, a recent CT assessment was not available). NFCB aetiology was idiopathic in 56%, postinfectious in 27%, TB-related in 8%, and of other causes in 9% of patients (postsurgical BE: 2.7%, DCP: 1.4%, hypogammaglobulinemia: 2.0%, Churg-Strauss syndrome: 0.7%, Kartagener syndrome: 0.7%, medium lobe syndrome: 0.7%, and occupational exposure: 0.7%).

Patient showed mild airway obstruction, no hyperinflation, and normal diffusing capacity; 24 patients had a bronchodilator reversibility greater than 12%. Frequent exacerbations (mean value, 2.5 exacerbations/year) were reported by 56% of patients, while persistent cough (20,4%), dyspnoea on exercise (11%), and sputum production and/or hemoptysis (6%) were reported in a limited number of patients. LCQ questionnaire showed a mild impairment due to cough, as only 21 out of 62 patients reported a score >0. NCFB severity was mild according to FACED and moderate according to BSI. Presence of airway pathogens was observed in 70.5% of patients, and of these 52% were represented by* Pseudomonas aeruginosa*. Patients with* Pseudomonas aeruginosa* infection showed lower levels of FEV1 in comparison with patients with other or no colonization (*P* = 0.007).

Regular pharmacologic treatment was performed by 82 patients (54.3%) with inhaled corticosteroids at different doses (always associated with long-acting beta2-agonist and/or antimuscarinic drugs) and by 71 patients (47%) with long-term oral azithromycin (750–1000 mg weekly). Relevant comorbidities were reported by 49 out of 152 patients (32%), in particular cardiovascular diseases (29%), upper airway diseases (26%), and GI diseases (13%).

Of 152 patients, 116 produced an adequate sputum sample. Inflammatory cell analysis showed a prevalent neutrophilic profile, with 69% of them having a sputum neutrophil percentage >64% [23]. Eosinophils percentages were equal to or more than 3% in only 20% of patients (Table 2). Patients with airway bacterial pathogens showed higher levels of sputum neutrophils than patients with no pathogens (*P* = 0.009), and those with* Pseudomonas aeruginosa* had even more sputum neutrophilia in comparison with those with other bacteria (*P* = 0.006). Mean levels of EBC-MDA and FeNO are also reported in Table 2. MDA levels were slightly higher than those previously reported in bronchiectasis (19.2 (6–54) nM) [21] and similar to those found in moderate COPD patients (29.1 (9–81) nM). Levels of FeNO >25 ppb were found in 25.5% of patients.

### 3.2. Correlations

FEV1% predicted correlated with BMI (*P* = 0.03; rho = 0.178) and number of pulmonary lobes involved in the disease (*P* = 0.039; rho = −0.21).

**Figure 1:**
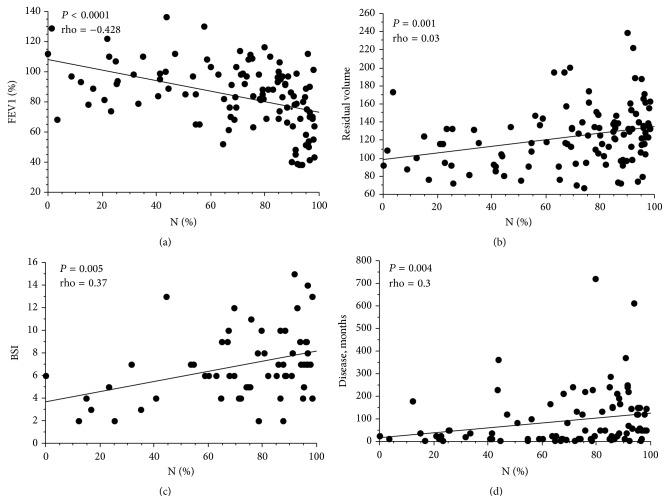
Correlation between neutrophils % in induced sputum and (a) FEV1, % predicted, (b) residual volume, (c) BSI, and (d) months between diagnosis of bronchiectasis and evaluation in the studied patients. Although these correlations are all statistically significant, the dispersion of the single data point is high.

Neutrophil percentages in induced sputum were inversely correlated with FEV1% predicted (*P* < 0.0001; rho = −0.428) and FEV1/VC (*P* < 0.0001; rho: −0.4), while sputum neutrophil percentages positively correlated with months between diagnosis and study evaluation (*P* = 0.004; rho = 0.3), residual volume (*P* = 0.001; rho = 0.03), and BSI severity score (*P* = 0.005; rho = 0.37), but not with FACED (Figure 1). Sputum neutrophil percentages were inversely correlated with FeNO (*P* = 0.01; rho = −0.28).

**Figure 2:**
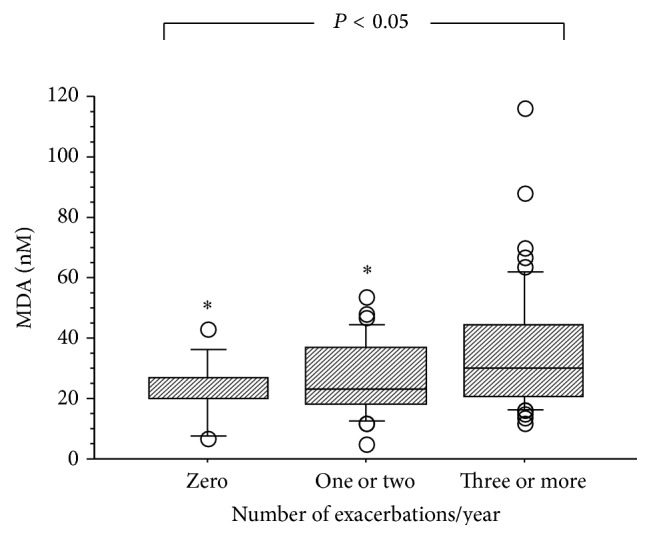
Box plots of malondialdehyde (MDA) in EBC according to the number of exacerbations in the preceding year. ^*∗*^ *P* < 0.05 by Dunn-Bonferroni post hoc test.

MDA levels in EBC significantly increased with the increasing number of exacerbations in the previous year (*P* < 0.05 by Kruskal-Wallis test) (Figure 2). In particular, patients with 3 or more exacerbations in the last year showed a significantly greater MDA concentration in EBC than patients with 1 or 2 exacerbations or patients without any exacerbations.

## 4. Discussion

In this study, the analysis of bronchial inflammatory biomarkers in induced sputum and exhaled air was aimed at a better characterization of adult patients with NCFB. We found that neutrophilia in induced sputum is a good marker of severity as confirmed by its correlation with several functional findings (FEV1, FEV1/VC, and RV), severity index (BSI), and symptoms (LCQ) assessment questionnaire and also with the time elapsed after diagnosis, suggesting that inflammation grows over time and might contribute to the progression of the disease. Moreover, neutrophilia was associated with the presence of airway pathogens, in particular with the presence of* Pseudomonas aeruginosa*.

The large majority of our patients (almost 70%) showed a percentage of sputum neutrophils greater than that observed in normal subjects [23], despite the fact that they were examined in a stable phase of the disease. This confirms that neutrophilic inflammation is a strong component of the disease. As reported by other authors [7, 24], sputum neutrophilia was associated with the type and severity of bacterial colonization, and also in our large group of patients, those with* Pseudomonas aeruginosa* had greater sputum neutrophilia and lower FEV1 in comparison with patients without airway pathogens or patients with other bacteria or fungi. In addition, we observed in 20% of our patients a sputum eosinophil percentage ≥3%, which is considered the upper limit of the normal distribution. To the best of our knowledge, this data has not been reported previously, and it can be due to the well-known association between bronchiectasis and asthma symptoms [25]. We did not find any relationship between the presence of sputum eosinophilia and acute FEV1 reversibility after salbutamol, clinical history of asthma, or treatment with inhaled corticosteroids (data not shown); other information regarding family history of allergic diseases, allergic sensitization, or bronchial hyperresponsiveness was not available in our patients. In any case, it is possible that this “asthmatic” component may play a role in the clinical manifestation of the disease and in the response to treatment.

Our study confirms and extends on a larger group of patients previous observations which have demonstrated the prominent neutrophilic systemic and airway inflammation in NCFB in comparison with control subjects and also some relationship between the level of inflammation and indices of severity of the disease, like pulmonary function, extension of bronchiectasis at HRCT, and bacterial colonization [4–8]. In addition to that, we found in our study new correlations not previously reported: in particular, sputum neutrophils directly correlated with residual volume, the duration of the disease, and its severity as assessed by the BSI score [14].

The inverse correlation between residual volume and sputum neutrophilia may suggest some involvement of small airways, leading to air trapping due to mucus plugging in the lower airways and then to a redistribution of lung volumes. This may be due to some potential extension of inflammation from airways of large or medium calibre to small airways, with potential consequences on the gas exchange.

We found a good correlation between sputum neutrophilia and duration of the disease, and also with BSI but not with FACED. In effect, BSI includes a larger number of variables than FACED and allows therefore a better stratification of the patients. In addition, FACED has not been validated and therefore it is uncertain whether it can be confidently used. To our knowledge, this is the first time that sputum neutrophilia has been related to this composite score assessing the severity of bronchiectasis, confirming that also this biomarker correlates with the comprehensive evaluation of disease severity as assessed by BSI.

Oxidative stress is a major component of the mechanisms leading to the damage of the airway wall in bronchiectasis [26] and also in other diseases, like COPD. In our study, MDA levels in EBC correlated with the number of exacerbations in the previous year. EBC-MDA concentrations in our patients were higher in comparison with a group of normal subjects studies in a previous methodologic paper [21]. MDA is a well-known biomarker of the oxidative stress, and we as well as other authors have demonstrated elevated MDA concentrations in EBC of patients with different inflammatory airway diseases [21, 27–29]. This means that MDA could have a role as indicator of future risk of exacerbation, potentially suggesting a different treatment strategy.

Our study has some limitations. Firstly, we did not include a control group of normal subjects; however, the difference between patients with NCFB and normal subjects as regards both systemic and airway inflammations is well known [4–8]. Secondly, we took into consideration only a single bacterial evaluation in the sputum for defining patients with bacterial colonization when repeated confirmations of the presence of sputum pathogens are required. In effect, in many of our patients, the presence of sputum pathogens has been confirmed in previous sputum specimens, and furthermore each patient was examined in a stable phase of the disease. Thirdly, some additional evaluations for explaining the presence of sputum eosinophilia in a subgroup of our patients were not included. Finally, we included both spontaneous and induced sputum in the inflammatory and microbiologic assessment; although these measurements are not interchangeable, previous studies have demonstrated similar inflammatory cell percentages in both samples [30]. On the contrary, this is one of the largest series of NCFB patients, examined in a very extensive way.

In conclusion, sputum neutrophilic inflammation in NCFB patients can be considered a good biomarker of disease severity, as confirmed by pulmonary function, disease duration, bacterial colonization, and BSI score.

## Figures and Tables

**Table 1:** Main clinical and functional data of the examined patients with NCFB.

Subjects, *n*	152
Male, *n* (%)	65 (42.7)
Age, years	62.6 ± 14
Smoking habit, yes/ex/no (%)	8/50/94 (5.3/32.9/61.8)
Pack/years	24.9 ± 18.8
BMI, Kg/m^2^	24.9 ± 4.8
FEV1, % pred.	84.0 ± 21.2
FEV1/VC, % pred.	84 ± 14
TLC, % pred.	104 ± 15
DLCO SB, % pred.	86 ± 18
LCQ, mean	15.0 ± 4.0
BSI, mean	7.1 ± 3.3
FACED score, mean	2.0 ± 1.5
Airway pathogens, %	
*Pseudomonas aeruginosa*	36.6%
*Staphylococcus aureus*	10.7%
*Aspergillus (gender)*	3.6%
*Non-TB mycobacteria*	1.8%
Other	17.8%
No colonization	29.5%

**Table 2:** Biomarkers measured in the sputum, EBC, and exhaled air in the examined patients with NCFB.

Sputum inflammatory cells	
Neutrophils, median (range), %	79.3 (1.5–98.1)
Eosinophils, median (range), %	0.8 (0–70.2)
Exhaled NO, ppb	22.5 (2–168)
MDA (EBC), nM	30.4 (6–116)
